# The effectiveness of digital multimedia presentation of trial information on recruitment and retention of patients: Protocol for a study within a trial (SWAT).

**DOI:** 10.12688/hrbopenres.12994.1

**Published:** 2020-03-30

**Authors:** Sinead Duane, Akke Vellinga, Valerie Smith, Marie Tierney, Claire Beecher, Megan Burke, Andrew W. Murphy, Declan Devane

**Affiliations:** 1 J.E. Cairnes School of Business & Economics, National University of Ireland, Galway, Ireland; 2 HRB Trials Methodology Research Network, National University of Ireland, Galway, Ireland; 3 Discipline of Bacteriology, National University of Ireland, Galway, Ireland; 4 Discipline of General Practice, National University of Ireland, Galway, Ireland; 5 School of Nursing and Midwifery, Trinity College Dublin, Dublin, Dublin, Ireland; 6 HRB Primary Care Clinical Trial Network Ireland, National University of Ireland, Galway, Ireland; 7 School of Nursing and Midwifery, National University of Ireland, Galway, Ireland

**Keywords:** Study Within A Trial, Recruitment, informed consent, primary care, Randomised Control Trial

## Abstract

**Background:** Studies within trials (SWATs) present an opportunity to examine design factors that may impact on the successful delivery of trials. One area in need of research is trial recruitment. Recruiting patients to trials is a major challenge facing trialists. Failure to meet recruitment targets can result in delays and underpowered studies. This SWAT evaluates the effectiveness of hand-held digital multimedia presentation of trial information and standard written patient information to potential participants on recruitment and retention to a host trial.

**Methods**: This is the protocol for SWAT 15, a two-group, embedded parallel randomised controlled trial (RCT) (ISRCTN12838042) designed within a host trial - the SATIN trial (ISRCTN88111427), a RCT designed for implementation in the Irish primary care setting. The SWAT eligibility criteria was determined by the host trial. General practices who agree to participate in the host trial will provide women (participants) who are willing to consider participating in the host trial with either a multimedia digital information resource facilitated through a handheld tablet device, plus a written participant information leaflet (Intervention) or a written participant information leaflet (comparator). Outcomes are recruitment and retention to the host SATIN trial and participant’s quality of decision-making.

**Discussion**: Although designed to be implemented in a host trial, the host trial, was suspended and therefore this SWAT was not implemented. The protocol and the lessons learnt whilst developing it offer guidance to researchers who wish to answer similar research questions in the future in a similar context or setting.

**Trial registration**: ISRCTN Registry ISRCTN12838042 (11/10/2017)

## Background

Rigorous research is essential to delivering and improving the quality of health care. Randomised controlled trials (RCT) provide reliable evidence on the benefits and harms of healthcare interventions ^1^. Although RCTs are accepted as the most appropriate method to evaluate the effects of health care interventions, there is strong evidence that recruiting clinicians and patient participants to trials presents a significant challenge ^2^. Across trials, it is estimated that less than 50% meet their recruitment target or do so only with an extension to the original trial duration ^3, 4^. For example, of 114 trials funded by the UK Medical Research Council (MRC) and the Health Technology Assessment (HTA) Programme that recruited participants between 1994 and 2002, only 31% met their recruitment targets and over half (53%) were given an extension ^5^. More recently, Sully and colleagues investigated the recruitment success of 73 trials funded by the same bodies between 2002 and 2008 and observed similar results. Only 55% of trials recruited to their pre-specified target sample size and nearly half (45%) received an extension ^6^. Similar issues have also been recognised in the United States. A study investigating the prevalence and associated economic impact of low-enrolling clinical studies at a single academic medical centre found that of the 837 clinical studies terminated during the study period, nearly a third (31.1%) were low-enrolling. Furthermore, primary care trials often fail to achieve adequate sample size as demonstrated in a recent study of primary care trials in which only 23% recruited successfully compared to 62% of mental health trials ^6^. These failures in meeting recruitment targets mean that overall trial findings are likely to be underpowered; with the studies delayed and falling short of answering their objectives ^3, 6^.

Understanding how to maximise the recruitment process will help to overcome these challenges in the future and would benefit trialists during the design and implementation phases of trials ^4^. Developing and evaluating interventions aimed at improving recruitment to trials may be a good investment, where even a small return could translate into avoidance of substantial additional costs whilst reducing the time to potential knowledge impact. However, there is, as identified in a recent Cochrane systematic review, limited high quality evidence evaluating the effectiveness of interventions to improve trial recruitment ^7^. Furthermore, over 35% (24/68) of trials included in this review evaluated the effectiveness of recruitment strategies to hypothetical trials meaning that the effectiveness of the strategies evaluated in real-life settings is further limited.

Methodological innovation is necessary to improve the science of recruitment and should be a focus when seeking to improve trial recruitment. The importance of establishing new methods of effective engagement among patients, practitioners, and the primary care research community is paramount; as there is a propensity among researchers to overestimate the degree to which research is viewed positively by practitioners and patients ^8^. It is acknowledged that one barrier to patient recruitment is inadequacy of trial information to meet the needs of potential participants as well as the ineffective mode of dissemination of this information. A failure to adequately explain what the trial is about, what participation involves, and the value of participation to potential participants has a direct impact on the informed consent process ^9^.

Studies Within A Trial (SWATs) have been developed as one method of gathering information on different design factors potentially impacting on the outcome of trials ^10– 12^. SWATs seek to *“aid the development of such research by increasing awareness of, and stimulating interest in the need for this research and providing a framework and resource to inspire and generate ideas, and to store, disseminate and modify such research”* ^13^. Researchers interested in conducting a SWAT are encouraged to register their SWAT in the SWAT repository.

This SWAT was registered as SWAT-15 in the SWAT repository. The host trial, SATIN (ISRCTN88111427); however, was stopped prior to recruitment of the first participant due to the emergence of new evidence on the treatment of urinary tract infections (UTI) and therefore this SWAT although designed was never implemented. The protocol and the lessons learnt whilst developing it could guide trialists who wish to answer similar research questions in the future. This protocol for SWAT-15, will be of use to researchers considering evaluating different ways to present information to potential trial participants and to those interested in SWATs in general.

## Aim

To evaluate the effectiveness of presenting potential trial participants with trial information using hand-held digital multimedia and written information leaflet or a standard written information leaflet, alone, on recruitment and retention to a host trial.

## Objectives

a) To establish if (a) the proportion of patients willing to consider participating and (b) the proportion of participants recruited to the host trial (in case this differs from the number of participants willing to participate, due to e.g. exclusion criterion) is improved using a hand-held multimedia presentation of trial information plus a standard written participant information leaflet compared to a standard written participant information leaflet, alone;
b) To explore whether a hand-held multimedia presentation of trial information plus a standard written participant information leaflet improves retention of patient participants to the end of the host trial;
c) To establish if the quality of decision-making as measured through a decisional scale, adapted from one used within the REFORM trial ^14^ and drawing conceptually on the SURE ^15^ and DelibeRATE scales ^16, 17^ is affected by the presentation mode (multimedia and written -v- written only) of participant information to patients.

## Methods

### Study design

A two-group, parallel embedded RCT using the SATIN trial as an example of how SWAT-15 could be implemented.

### Study population

The study population will be individuals who will be screened for and/or who are eligible to take part in the host SATIN trial.

***Inclusion criteria***

To participate in SWAT-15 individuals must, as determined by the host SATIN trial:

- Be attending a general practice that is taking part in the trial;
- Have a GP-diagnosed UTI, and at least one of the symptoms of dysuria, urinary frequency, or urgency with/without low abdominal pain;
- Be a woman (non-pregnant) aged 18 years or above;
- Be able and willing to give written informed consent;
- Own a smartphone.

***Exclusion criteria***

Exclusion criteria are as per the SATIN trial i.e., any signs of complicated infection or any condition that may lead to complications, current or recent antibiotic use, recent UTI, current intake of NSAIDs, pregnancy or breastfeeding, non-use of highly effective contraception, previous adverse reaction to any of the study drugs, current intake of drugs potentially interacting with the trial drugs, diabetes mellitus, chronic kidney disease or any other previous illness related to kidney or urinary tract, history of gastro-intestinal ulcers, Glucose 6 phosphate Dehydrogenase deficiency or any other medical condition that may put the participant at risk or influence the study results in the investigators’ opinion.

### Study setting

The SWAT will be carried out in general practices.

### Assignment of interventions

Allocation will be performed at the individual patient level with half of the participants receiving:

a) Multimedia digital information resource facilitated through a handheld tablet device, plus a written participant information leaflet, within the general practice (n=230) (Intervention) (see extended data ^18^)
  OR
b) Written participant information leaflet (n=230) (Comparator) (see extended data ^19^)

A cluster design could also be utilised but the resultant impact on study power would need to be considered. During the study period, potentially eligible participants (n=460) will be identified by the GP during their routine consultations based on the inclusion and exclusion criteria outlined above. Patients who are deemed eligible and willing to consider participating in the host study will be seen by a practice nurse or General Practitioner (GP). The practice nurse or GP will give consecutive eligible patients the next sequentially ordered, participant information leaflet, which will be taken from the top of the bundle of trial information pack. Attached to the participation information leaflet will be a sequentially numbered, sealed, opaque envelope with an ‘envelope ID’ number on it and a card inside with details of the woman’s group allocation. Group allocation will be determined by computer generation of a random allocation sequence with a 1:1 ratio and block sizes of 4, 8 and 8 (at random). As per allocation, the practice nurse or GP will give the potential participant either the patient information leaflet with a handheld tablet device and headphones to access the multimedia information (intervention) or no additional information i.e. the potential participant is given an information leaflet only (comparator). The practice nurse or GP will subsequently record the envelope ID number on the SATINs screening and enrolment log. The GP and/ or practice nurse will have been trained in the requirements of the host trial, and this SWAT, including in the use of the digital resource.

### Sample size, estimated effect size and power

Primary analysis is the comparison of the differences in proportions of women recruited to the SATIN trial between intervention and comparator. The host trial aims to recruit 460 women. Based on a background proportion recruitment (i.e., randomised) of 64% of eligible women agreeing to participate in the SWAT using conventional written patient information (only) ^20^ and an acceptable error rate of α = 5%, this SWAT will have power of 80% to detect a 18% relative increase in recruitment proportions between control and intervention groups (i.e., 64% v 76%, absolute difference 12 percentage points).

We acknowledge that the effects we are assuming are relatively large for a test of two different ways of presenting trial information to potential participants. However, the sample sizes of SWATs addressing important methodology questions are limited by the size of the host trial. SWATs provide important data for appropriate pooling and meta-analysis, and the evidence base can be developed by encouraging other trials to run the same intervention in other contexts. This study will add to the limited evidence base in the area of trial recruitment and enable the development of pooled datasets capable of informing whether intervention effects vary by country, trial, or participant population.

### Designing recruitment material

Both comparator and intervention arms focus on the provision of trial information to eligible participants. The decision to focus on presentation of patient information was based on the limited amount of empirical evidence available on how the quality of patient decision making is effected by the use of multimedia patient information and whether different modes of presentation can improve recruitment ^21^. Both trial arms will provide similar information and will conform to Good Clinical Practice guidance ^22^ and to the Declaration of Helsinki ^23^ for gaining informed consent.

### Comparator: Written participant information leaflet

Participants in the comparator arm will receive written participant information. The design was informed by examples of similar patient information leaflets and the requirements of Good Clinical Practice ^22^. Information provided within the written participant information leaflet answer each of the following questions:

- Can I stop taking part if I wish?
- What is the purpose of this study?
- Why is this study important?
- Why have I been asked to take part in this study?
- What does taking part mean for me?
- What will I be asked to do?
- Is my information confidential?
- What are the benefits of taking part?
- What are the risks of taking part?
- What do I do if I feel worse or do not improve?
- What happens if I suffer complications because of the study?
- Compensation
- Who should I contact if I’m concerned about the running of this study?
- Where can I find more information?
- Further queries

The content of the written participant information leaflet was reviewed by a practice nurse, host trial steering group members, the host trials Public and Patient Partnership in Research (PPP-R) group and the Health Research Board Clinical Research Facility, Galway, (HRB CRFG) representatives, who suggested changes to the content. The literacy level of the participant information leaflet was assessed using readability formulas (SMOG) available online ^24^.

### Intervention design

The design of this SWAT intervention was informed by best practice approaches demonstrated by the MRC START (Medical Research Council Systematic techniques for assisting recruitment to trials) programme of recruitment research ^25^. The MRC START programme of recruitment research was developed based on relevant theoretical and empirical work about patient decision-making generally and in trials specifically. Our SWAT intervention design was also informed and constrained by the SATIN trial design and the complexity of the general practice setting in which we proposed it would be implemented. The multimedia digital information resource needed to be easily accessible through an electronic tablet device which will be provided to the potential participants within the general practices and provide adequate information in a relatively short period of time (e.g. 15–20 minutes). The researchers sought to make the resource as sustainable as possible therefore integrated the content into the same pre-existing website, which also supported the host trial support material.

The MRC START programme adopted a process for optimising readability and navigation of participant recruitment material ^25, 26^. A similar approach was adopted to design this SWAT intervention. The SWAT-15 core team members combined their expertise with findings from previous published trial recruitment research ^3, 4^, patient decision making research ^27^, and behavioural theory. The structure and content of the multimedia digital information intervention was informed by PPP-R forum members feedback, a steering group consisting of international experts in trial recruitment, and by the generic website template provided by the MRC START team ^28^. The written participant information leaflet (comparator) formed the basis for the development of this multimedia digital information intervention.

**Figure 1.**
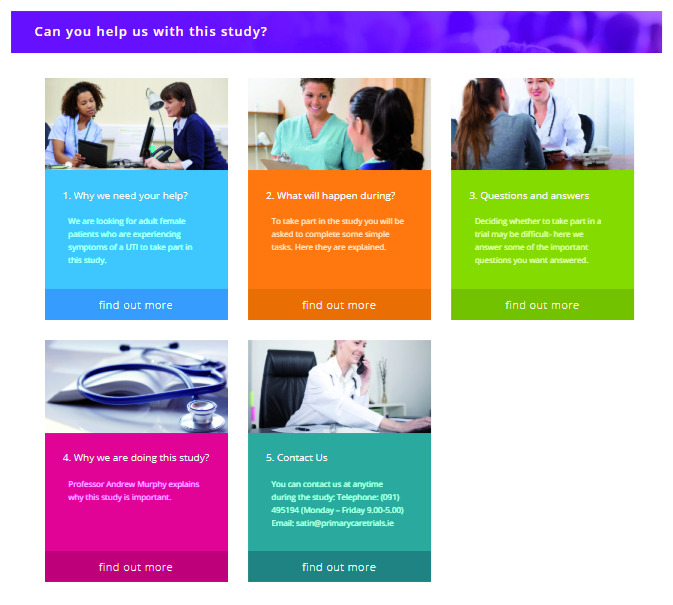
Website template.

The multimedia intervention component was made up of six sections, which repeat the written patient information in text format and supplemented it with multimedia resources ( Figure 1).

1. Why we need your help?- A video from the lead researcher describing the rationale for the trial (trial specific)
2. What will happen during the study?- An infomercial explaining what the patient was expected to do during the trial (trial specific)
3. Questions and Answers
4. Why are we doing this study?
5. What are randomised controlled trials? - An infomercial describing what random allocation is (universal generic information)
6. Contact Us

The universal generic applicable content related to trial design was adopted from the MRC START resource ^25^.

During the design phase, the research team drafted an outline of the content of the multimedia website and wireframes of the proposed content and website navigation. The scripts for the generic and host trial video and infomercial were drafted; the text to accompany the videos came from the comparator patient information leaflet.

A ‘think aloud method’ was used to test the design and content of the website. This technique has been used previously to improve patient versions of clinical guidelines ^29^. The ‘think aloud’ method uses semi structured interview guide to explore first impressions of the multimedia digital information ^30^. The five semi structured interviews conducted explored six facets for the user experience namely credibility, usefulness, desirability, findability, and value ^31^. The seventh facet of Morville’s model, accessibility, was also explored as the intervention needed to be easily accessible on a tablet device ^32^. The process of integrating the multimedia digital information through a handheld tablet into practice will be pre-tested in one general practice prior to the launch of the host RCT. During this process, additional elements such as ease of use and the instructions of how to use the device will be tested and changes made as required.

### Informed consent

Informed consent will be sought for all women who decide to participate in the host trial, which was granted ethical approval from the Irish College of General Practitioners Ethics Committee (1 ^st^ December 2016). Formal consent to participate in the SWAT, at the individual, patient-participant level will not be sought as this embedded study is not withholding information; instead the focus is on how it is presented. All potential participants will receive the host trial participant information leaflet approved by the Research Ethics Committee. Further, by telling participants that they are being randomised to different recruitment strategies would not only contaminate the results and undermine the intervention being tested, as it would unduly draw potential trial participant’s attention to the recruitment process, it would introduce complexity and likely confusion for participants due to the double consent present. There is precedence for this, as a similar approach has been used previously within the MRC START programme of research on the basis that the embedded study is not withholding information – just changing the way it is presented (while also presenting in traditional information leaflet format) (NRES Committee Yorkshire and the Humber – South Yorkshire (REC Reference 11/YH/0271)). Permission to proceed with the current study, without obtaining formal consent, was approved by the ICGP Ethics Committee (1st December, 2016).

### Outcomes

***Primary outcome***

The primary outcome will be proportion of potentially eligible participants willing to participate in the host trial.

The number of eligible participants willing to consider participation (numerator) will be calculated as the number of participants who sign the host trial informed consent form (including those that are subsequently found not to fulfil the eligibility criteria). The denominator will be the number of potential participants who are randomised to receive either the intervention or comparator.

***Secondary outcomes***

a) The proportion of participants recruited to the host trial (in case this differs from the number of participants willing to participate, due to, for example, an exclusion criterion).

This will be recorded in the host trial electronic case report form (eCRF) (or equivalent). The host trial research team will share the number of recruited participants to their study (i.e., the number of participants who sign a consent form and complete screening through the eCRF) with the SWAT research team. Demographic information (i.e., age, education, number of children, health insurance status) will be collected as part of the host study from participants who give informed consent and shared with the SWAT team. All data will be aggregated and anonymous and it will not be possible for the SWAT research team to identify individual participants.

The nurse/ GP will complete a screening and enrollment log for all patients who are willing to consider participating in the study. Any patient who receives the study intervention or comparator will be allocated an envelope ID number and this will be recorded on the screening and enrollment log. The envelope ID number is the key to identifying the intervention to which participants were randomised.

The practice nurse will also record the patient ID number on the screening and enrollment log form. A patient ID is generated within the eCRF when it is opened and the patient has given informed consent to participate. The patient ID will allow the SWAT team to identify if the demographic characteristics differ between consenting patients in the intervention and comparator groups.

b) The proportion of recruited participants who are retained to the end of the host trial;

Retention will be measured as the number of participants who complete outcome measures in the host trial.

c) The quality of decision-making

Quality of decision-making will measure the women’s understanding of participating in the host trial. The quality of decision-making by potential host trial participants will be measured through the completion of a decisional scale, adapted from one used within the REFORM trial ^14^ and drawing conceptually on the SURE ^15^ and DelibeRATE scales ^16, 17^. The SWAT research team will analyse these data after the host study has finished recruiting and the last patient has completed the study.

### Statistical analysis plan

Descriptive statistics and correlations will be reported. The analysis plan will be as per stated in the study outcomes. The number of eligible participants willing to consider participation (numerator) will be calculated as the number of participants who sign the host trial informed consent form (including those that are subsequently found not to fulfil the eligibility criteria). The denominator will be the number of potential participants who are randomised to receive either the intervention or comparator. Number of patients retained will be calculated as a frequency.

### Data management plan

The Data Management Plan was developed alongside the SATIN trial. If central unblinding for the host trial is necessary this will be provided by a specialised external company. All recruitment and consent data will be managed by the SATIN team as part of the host trial. The host trial will give the researchers access to anonpymised information in relation to recruitment and retention to the host trial. Data will be captured on an encrytpted electronic CRF and a survey included in the SATIN mobile App.

### Dissemination of information

The findings of this SWAT were to be published in Peer review journals and presented at both international and national conferences. As this SWAT was terminated prior to recruitment, it is hoped that this protocol could be implemented in another setting.

### Study status

The host trial, SATIN ( ISRCTN88111427); was stopped prior to recruitment of the first participant due to the emergence of new evidence on the treatment of urinary tract infections (UTI) and therefore this SWAT although designed was never implemented.

## Discussion

Improving the efficiency of how randomised trials are planned, conducted, analysed and reported is an important area of research and one of increased interest by the trial community ^33^. Inadequate recruitment to trials has been identified as an important contributor to research waste that contributes to increased research costs and delayed information on the effectiveness of health care interventions ^3, 4^.

This protocol for SWAT-15 offers an opportunity to answer important questions on efficiencies in trial processes by embedding primary trial methodological studies within host randomised trials. This SWAT has the potential to evaluate the effectiveness of a handheld digital multimedia presentation of trial information and written participant information to potential participants on recruitment and retention to the host SATIN trial. This will help inform future recruitment and retention strategies to trials evaluating Investigational Medicinal Products in the primary care setting.

### Ethical approval

Ethical approval was granted by the ICGP Ethics Committee on 1st December 2016

## Data availability

### Underlying data

No data are associated with this article

### Extended data

Figshare: SWAT Patient Information Leaflet. https://doi.org/10.6084/m9.figshare.11894385.v2 ^19^

This project contains the following extended data:

- SATIN PIL Version 4.0 08062017.pdf (Patient information leaflet)

Figshare: SWAT Website. https://doi.org/10.6084/m9.figshare.11923146.v1 ^18^

This project contains the following extended data:

- SWAT Website.pdf (Series of screenshots of the SWAT website)

### Reporting guidelines

SPIRIT checklist for ‘The effectiveness of digital multimedia presentation of trial information on recruitment and retention of patients: Protocol for a study within a trial (SWAT).’ https://doi.org/10.6084/m9.figshare.11894355.v1 ^34^
