# Supplementary material for: The effectiveness of digital multimedia presentation of trial information on recruitment and retention of patients: Protocol for a study within a trial (SWAT)
Source: HRB Open Res. 2020 Mar 30;3:10. [Version 1] doi: 10.12688/hrbopenres.12994.1 (PMC7308906; doi:10.12688/hrbopenres.12994.1)
Supplement: Peer review history

# Peer review: Reviewer response for version 1

**DOI:** 10.21956/hrbopenres.14084.r27499

**Published:** 2020-06-22

**Authors:** Frances Shiely

**Affiliations:** 1 School of Public Health, University College Cork, Cork, Ireland; 2 HRB Clinical Research Facility at UCC, Mercy University Hospital, Cork, Ireland

This is a protocol for a SWAT that was not implemented because the host trial was stopped before recruitment began. The researchers argue, rightly so, that this protocol is still important to others who may wish to contribute to improving recruitment in clinical trials, which has been cited in this protocol as a key issue of concern for trialists. The aim of the SWAT was evaluate the effectiveness of presenting potential trial participants with trial information using hand-held digital multimedia and written information leaflet or a standard written information leaflet, alone, on recruitment and retention to a host trial. Though the host trial this was initially written for was for a trial in a primary area setting, this protocol is applicable to trials in any setting with tweaking to the inclusion and exclusion criteria which will be host trial dependent.

**Is the rationale for, and objectives of, the study clearly described?**

 Yes, the rational and objectives are clearly described.

**Is the study design appropriate for the research question?**

 Yes the study design is appropriate, but I have queries on the section related to the sample size, estimated effect size and power. The authors calculate a sample size, and state that the effects they are assuming are large. I question performing a sample size calculation in this SWAT. We know that most SWATS are underpowered and are designed with future meta-analysis in mind (Treweek *et al*. 2018) ^1^. On the one hand, the authors acknowledge that the sample size of the SWAT is limited by the size of the host trial, and they state that SWATs provide important data for pooling and meta-analysis, but they still go on to make the sample size calculation, based on a large effect size. A line or two to explain the reasoning behind calculating a sample size for the SWAT in a relatively small trial, would be helpful. 

**Are sufficient details of the methods provided to allow replication by others?**

 Yes, the methods are clear and will allow the study be conducted by others.

**Are the datasets clearly presented in a useable and accessible format?**

 Not applicable.

**Conclusion**

 This is a well-designed SWAT that will contribute to the evidence base on trial recruitment methodology. As an aside, one aspect of this SWAT that could be explored by researchers is the Ethical approval to conduct the SWAT without separate SWAT consent. In my experience, this is not standard and is something that needs exploration and clarification in the literature.

Is the study design appropriate for the research question?

Yes

Is the rationale for, and objectives of, the study clearly described?

Yes

Are sufficient details of the methods provided to allow replication by others?

Yes

Are the datasets clearly presented in a useable and accessible format?

Not applicable

Reviewer Expertise:

Epidemiology; Clinical Trial Methodology; SWATs.

I confirm that I have read this submission and believe that I have an appropriate level of expertise to confirm that it is of an acceptable scientific standard.

## References

[1] : Trial Forge Guidance 1: what is a Study Within A Trial (SWAT)?. *Trials*.2018;19(1) : 10.1186/s13063-018-2535-5 10.1186/s13063-018-2535-5 29475444PMC5824570

# Peer review: Reviewer response for version 1

**DOI:** 10.21956/hrbopenres.14084.r27318

**Published:** 2020-04-27

**Authors:** Bronagh Blackwood

**Affiliations:** 1 The Wellcome-Wolfson Centre for Experimental Medicine, Queen's University Belfast, Belfast, UK

This is an excellent protocol for a SWAT designed to maximise recruitment and retention using a multimedia presentation of the participant information sheet (PIS) versus the written PIS. The design of the multimedia presentation, based on best practice, is very well described. I have only three comments.

1. I would recommend a little more clarity around the presentation of the interventions to potential participants to show the time taken for delivering both interventions. For the comparator PIS, apart from giving the PIS to participants, will the practice nurse/GP discuss this with participants? If so, approximately what amount of time will this take? For the intervention group that receive both PIS and multimedia, will the practice/nurse also discuss the PIS (if this is done for the comparator group) or is the 15-20 minute multimedia designed to replace that discussion? So for the intervention group, what approximate total amount of time would this take?
2. Is there a mechanism for recording both practice nurse/GP and participant adherence to the allocated intervention as this may be influenced by the time needed to present both interventions? For example, in the intervention group, if the PIS is provided, but the participant doesn’t wish to spend the time, or doesn’t have the time, to go through the multimedia programme.
3. Following on from that, will the statistical analysis be according to intention to treat or as per intervention received?

Is the study design appropriate for the research question?

Yes

Is the rationale for, and objectives of, the study clearly described?

Yes

Are sufficient details of the methods provided to allow replication by others?

Partly

Are the datasets clearly presented in a useable and accessible format?

Not applicable

Reviewer Expertise:

My area of clinical expertise is critical care; my methodological expertise includes clinical trials, systematic reviews and process evaluations of trials.

I confirm that I have read this submission and believe that I have an appropriate level of expertise to confirm that it is of an acceptable scientific standard.
